# Improved profile HMM performance by assessment of critical algorithmic features in SAM and HMMER

**DOI:** 10.1186/1471-2105-6-99

**Published:** 2005-04-15

**Authors:** Markus Wistrand, Erik LL Sonnhammer

**Affiliations:** 1 Center for Genomics and Bioinformatics, Karolinska Institutet, S-17177 Stockholm, Sweden

## Abstract

**Background:**

Profile hidden Markov model (HMM) techniques are among the most powerful methods for protein homology detection. Yet, the critical features for successful modelling are not fully known. In the present work we approached this by using two of the most popular HMM packages: SAM and HMMER. The programs' abilities to build models and score sequences were compared on a SCOP/Pfam based test set. The comparison was done separately for local and global HMM scoring.

**Results:**

Using default settings, SAM was overall more sensitive. SAM's model estimation was superior, while HMMER's model scoring was more accurate. Critical features for model building were then analysed by comparing the two packages' algorithmic choices and parameters. The weighting between prior probabilities and multiple alignment counts held the primary explanation why SAM's model building was superior. Our analysis suggests that HMMER gives too much weight to the sequence counts. SAM's emission prior probabilities were also shown to be more sensitive. The relative sequence weighting schemes are different in the two packages but performed equivalently.

**Conclusion:**

SAM model estimation was more sensitive, while HMMER model scoring was more accurate. By combining the best algorithmic features from both packages the accuracy was substantially improved compared to their default performance.

## Background

Computational protein sequence homology detection has become a central component in genome analysis. Today sequences of unknown function are routinely searched against databases of known proteins and this has become an important aid for sequence annotation and to guide laboratory experiments. Without the development of software tools for the detection of protein homology from amino acid sequence this would not have been possible. Such homology detection tools aim to find similarities between related proteins and to score them above the noise level. Different methods have varying degrees of success and it has been shown that profile-based methods, which consider information from a number of sequences, perform better than pairwise methods[1]. In particular, profile Hidden Markov models (profile HMMs)[2,3] have generated good results, and are today employed by several databases. Pfam[4] and Superfamily[5] for example, are large collections of protein families where each family is represented by a profile HMM. The profile HMM is a probabilistic model of a multiple sequence alignment of the family and is used to represent the family in database searches.

Several aspects of profile HMM technology have been further developed since its initial conception. Various schemes for sequence weighting have been proposed[6,7] and different null models have been studied[8]. The introduction of Dirichlet mixtures to model prior distributions[9,10] constituted a major step forward. The maximum likelihood technique employed to estimate such prior distributions has, however, been shown to be problematic for transition priors[11]. Discriminative training has been incorporated into model building and been shown to give improved performance[12,13]. Methods that incorporate phylogenetic information directly into the profile HMM and bypass the need for ad-hoc sequence weighting, have been developed and proved promising for homology detection [14,15]. Secondary structure prediction has been combined with profile HMMs into a probabilistic framework for more accurate fold recognition[16,17]. Finally, explicitly including knowledge about the taxonomic distribution of protein domains has proved to enhance protein domain discovery[18], as has the incorporation of knowledge about the likelihood of certain domain combinations[19]. These examples of HMM improvements are only a few and by no means a complete listing.

Two widely used profile HMM packages are SAM[2] and HMMER[3]. It is of interest for users to know the relative performance of the programs, and for profile HMM developers to know the key factors for good performance. Madera and Gough contributed the most recent and still most thorough comparison of the two programs[20]. The authors divided their analysis into the two main steps of profile HMM homology detection: model building and database searching. Model building involves converting a multiple alignment of the family into a probabilistic model, while database searching involves scoring a sequence to the profile HMM. The two steps are independent and by using a program to convert HMMER models into SAM format and vice versa, Madera and Gough were able to separately evaluate the building and scoring performance of the two programs. SAM model building was found to be clearly superior to HMMER model building, while no conclusion could be made concerning the scoring algorithms. E-value calculation, low complexity masking and time consumption were also investigated, but neither of the packages stood out as clearly better than the other.

Profile HMMs often model complete protein domains while real proteins may contain several domains. It therefore makes sense to look for a local match of the protein to the HMM. "Global/local matching" forces the entire HMM to match a part of the sequence. This is often the desired mode if the HMM is built from one domain and the entire domain can be expected also in other proteins, perhaps in combination with other domains. In contrast, "local/local matching" means that a part of the HMM is matched locally to the sequence. The choice between the two modes depends on the application. Local/local searches can find fragmentary protein sequences that would get poor scores if they were forced to match the entire model. However, in case the query sequences contain full domains, the sensitivity of the global/local mode should be better.

Madera and Gough compared the packages only for local/local mode. The first of the two objectives of this article is to extend their analysis to global/local searches. The second objective is to find the key features for profile HMM performance with a particular focus on model building. Profile HMM estimation involves choices concerning for example sequence weighting, prior probabilities, and model architecture, and the two programs approach these issues differently. By comparing SAM and HMMER run with non-default settings and with parameters borrowed from each other, we show which model parameters are crucial for profile HMM performance.

The article has the following structure. First we introduce the SAM and HMMER packages and explain the role of the model building components that we will investigate. Second we go through the test procedure, the data sets and the performance measure that we use. In the result section we compare the packages and analyse the influence of algorithmic components and parameters on the HMM performance in terms of sensitivity and specificity.

## Profile HMMs – HMMER, SAM and relevant parameters

The SAM package comes from the University of California Santa Cruz. The package includes the SAM-T2K iterative procedure to generate multiple alignments and HMMs starting from a single sequence[21]. Another feature is "multi-track HMMs", to process more than just the primary sequence data. Secondary structure information can, for example, be incorporated in a probabilistically sound way for better modelling[17,22]. This article will not evaluate these two additional features (both lacking in HMMER), but deals with traditional profile HMMs built from multiple alignments. The SAM package is used by the SUPERFAMILY[5] database. HMMER is developed by Sean Eddy and is open-source, well documented and easy to use. HMMER and the protein family database Pfam have co-evolved, but today HMMER is the engine also in other databases, including TIGRFAMs[23] and SMART[24].

In this study we used SAM version 3.4 and HMMER version 2.3.2.

### HMM architecture and construction

A profile HMM is a probabilistic model of a multiple alignment of related proteins. The alignment is modeled using a series of nodes (roughly one per alignment column) each composed of three states: match, insert and delete. Match and insert states emit amino acids with probabilities learned during model estimation while delete states are quiet. Insertions and deletions with respect to the HMM are modeled by insert and delete states and transition probabilities to them. The original profile HMM architecture allowed transitions between all states, which gives 3 × 3 = 9 possible transitions for each node. SAM has kept this architecture, while HMMER since version 2.0 allows seven transitions only. In the HMMER architecture transitions from insert to delete states, and vice versa, are forbidden.

Both HMMER and SAM allow the user to "label" columns in the multiple alignment to tell the program which columns should be seen as match/delete states and which should be seen as insert states. In case such information is missing, SAM will assign every column to a match state and produce an HMM with one node per column. In HMMER an algorithm will assign columns to match or insert states so as to maximize the posterior probability of the aligned sequences, given the model. Compared to SAM, this normally results in shorter models since some of the columns are assigned to insert states.

### Prior probability alternatives

Profile HMM parameters are estimated by combining the observed data (the multiple alignment) and a prior over probability distributions. If the multiple alignment were a good representation of the underlying protein family, there would be less need for using a prior. However, this is often not the case, primarily because the alignment includes too few sequences. The strength of priors is in compensating for small sample size and to distribute probability also to unseen events.

SAM and HMMER both use Dirichlet mixtures to model emission prior probabilities[9,10]. A Dirichlet mixture consists of a number of Dirichlet components, which are distributions over probability parameters. Each component typically captures a specific feature of columns observed in multiple sequence alignments, i.e. hydrophobicity or polarity, but also the degree of conservation to certain amino acids. During model building, the Dirichlet components are weighted probabilistically for each column in the multiple alignment (optimally given the amino acid frequencies of the column) and combined with the observed amino acid frequencies to obtain the posterior emission probabilities. The default emission prior in SAM is currently a mixture of 20 components, while HMMER's default is a mixture of nine components. Transition prior probabilities are modeled by single distributions in both SAM and HMMER, but differ in two ways. First because SAM employs nine parameters and HMMER only seven, i.e. one per transition. Second because SAM assigns a higher prior probability to deletions and insertions than HMMER.

### Sequence weighting: relative and total weights

The weight assigned to a sequence determines its influence on the final HMM. Without sequence weighting, a high redundancy among the sequences would make the model skewed and it would not recognize under-represented sequences. The sequence weights are calculated in a two-step process: relative weights are first determined and then scaled to sum to the total weight ("effective sequence number" in HMMER terminology), which is calculated separately.

The relative weights determine the influence of one sequence relative to the others. There are several algorithms for relative sequence weighting and common for all of them is to assign less weight to similar sequences and more to the divergent but still trusted family members. The HMMER default algorithm derives weights from a sequence tree relating the sequences[25], while SAM uses an unpublished algorithm based on relative entropy (SAM documentation). Relative weights do not sum to any particular value, but are scaled to add up to the total weight. The total weight thus governs the weight of the multiple sequence alignment relative to the prior probabilities. HMMER and SAM calculate the total weight in two very different ways. HMMER applies an algorithm that groups sequences by single-linkage clustering and counts the number of clusters above a specified level of identity. SAM scales the weights according to an entropy target that specifies the number of bits per column to save during model building, i.e. the information content of the final model compared to a background model.

### Global or local scoring

A sequence can be scored locally to the entire profile HMM (global/local) or to a part of it (local/local). In HMMER, the search mode is specified in the HMM at the time of model building. Two HMMs can thus be built from the same alignment, one global/local and one local/local, and both are scored using the same algorithm. SAM estimates only one HMM for each multiple alignment, and the search mode is instead specified at the time of model scoring.

## A SCOP/Pfam based benchmark

### Data sets

A large number of studies have used the SCOP[26] structural classification for evaluating the performance of sequence homology detection methods[13,18,20,27,28]. SCOP is a database classifying all protein domains of known structure into a hierarchical order of four levels: class, fold, superfamily and family. Two domains belong to the same family if they have a clear common evolutionary origin revealed either by a minimum of 30% sequence identity or very similar structure and function. Two domains belong to the same superfamily but different families, if a common evolutionary origin is not obvious from sequence identity, but probable from an analysis of structure and functional features. The fold level is grouping all superfamilies and families that have a common pattern of secondary structure elements. Finally, the class level divides domains into large classes based on secondary structure components.

In this work we evaluated the performance of profile HMMs for homology detection at the superfamily level. We wanted to avoid conditioning the results on the use of a particular program to generate the multiple alignment. Following Coin et al[18], we therefore developed a test set that combines the high quality Pfam alignments and the SCOP classification. Pfam is a database of protein families based on sequence similarity rather than structural similarity. A manually curated sequence alignment is provided for each family, as is a profile HMM to search for homologs. We used the ASTRAL data set filtered to a maximum of 40% sequence identity for the SCOP sequence classification[29]. ASTRAL is a database derived from SCOP and provides sequence files filtered to various levels.

To generate the test dataset, Pfam families were linked to the superfamily level in the SCOP classification. We kept all Pfam families that contain sequences that belong to one and only one SCOP superfamily. We also required that the SCOP domain definition for at least one of the sequences spanned the entire Pfam domain. Using Pfam 15.0 and the ASTRAL dataset this gave a test set of exactly 1400 families. We imposed two extra conditions: that the Pfam seed alignment had at least 10 sequences and that the average sequence length was above 30 amino acids. This gave 1009 families from which we extracted every second family in alphabetical order to get a large enough but yet computationally feasible set of 505 families. All in all, the dataset contains 9 411 positive and 2 842 994 negative test sequences.

### Test procedure

The test procedure was the following. We built profile HMMs from the seed alignments of the 505 Pfam families. We scored the entire ASTRAL dataset to the HMMs and classified the matches from their SCOP classification. Matches to the SCOP superfamily mapped to the query Pfam family were classified as true hits. Matches to a different SCOP fold were classified as false hits, while matches to the same SCOP fold as the query but a different superfamily were ignored. For each HMM, the searching generated a list of hits that we sorted on E-values. We went through this list from top to bottom and for each level of false positives we recorded the number of true positives. This gave a plot of true positives versus false positives, which is the standard way of displaying results from this type of tests.

## Results and discussion

### Default settings

**Figure 1:**
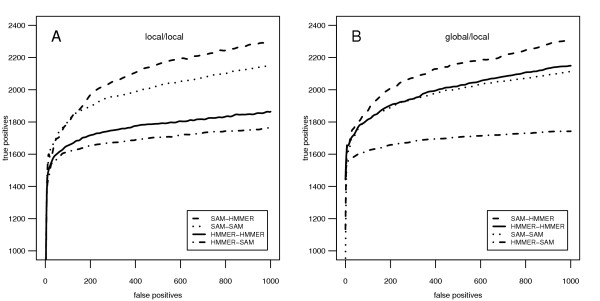
**Model building and database searching performance of SAM and HMMER**. **(A) **Local/local mode. **(B) **Global/local mode. The Viterbi algorithm was used for all searches. Otherwise, default settings were used. The model building program is mentioned first in the legend, and the scoring program second. 'SAM-SAM' means 'Building the HMM using SAM; Searching using SAM', 'HMMER-SAM' means 'Building the HMMs using HMMER; Searching using SAM', etc.

SAM and HMMER were compared for both local/local and global/local mode. We first ran the packages in local/local mode using all default settings, except that SAM scoring was performed by the Viterbi algorithm. Figure 1a shows that SAM performed considerably better than HMMER; building and scoring using SAM detected more true positives than building and scoring using HMMER, and this was true across all error rates. However, the best results were obtained when SAM models were converted to HMMER models and scored by HMMER. In contrast, HMMER models converted to SAM models followed by SAM scoring produced the worst results. It is thus fair to say that model estimation is considerably better done by SAM, while scoring is better done by HMMER.

Figure 1b shows the corresponding results for global/local mode. Here SAM and HMMER produced nearly identical results and no conclusion could be drawn as to which is the better package. Splitting the performance into building and scoring, it became evident that HMMER scoring was the best choice for both HMMER and SAM models. Consequently, SAM's model building was more accurate than HMMER's. In agreement with local/local mode, SAM model estimation was superior, but for global/local mode this advantage was fully compensated by HMMER's more accurate scoring program.

It is striking how much worse HMMER performed in local/local mode compared to global/local. In contrast, the SAM results for local/local mode were very close to those for global/local mode. Remember that the two packages have solved the issue of global or local scoring in different ways: while HMMER has two separate models and one way of scoring, SAM has one model and two ways of scoring. Could it be that the HMMER local/local model architecture, rather than the actual parameter estimation, is causing the poor local/local performance? If this were the case, SAM models converted to HMMER format and configured for local/local scoring should be less accurate than SAM models converted to HMMER format and configured for global/local scoring. This was not the case in our test (compare the SAM-HMMER curves in Figure 1a and 1b). Instead, the reason must be poorer model building in HMMER than in SAM, affecting local/local models more than global/local models.

To conclude this section we note that although HMMER proved comparable to SAM for global/local scoring, SAM is the preferred package as it performed much better in local/local mode. SAM produced better models, but lost some of the advantage due to an inferior scoring program. While HMMER model building was underperforming overall, local/local models proved particularly poor. In what follows we will seek explanations to these differences by analysing the effect of relevant model estimation parameters and algorithmic choices.

### Prior probability options

**Figure 2:**
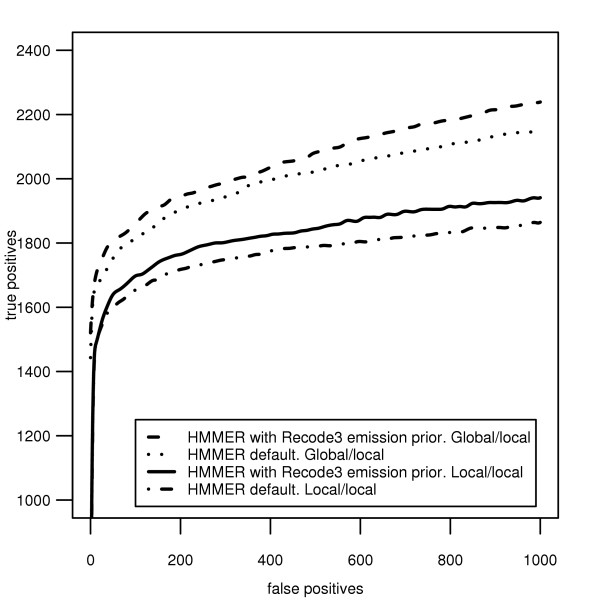
**Analysis of emission prior probabilities**. HMMER models were estimated using the SAM emission prior recode3.20.comp. Both local/local and global/local models were improved compared to using the HMMER default emission prior. Part of the SAM – HMMER performance difference can therefore be assigned to the emission prior.

The default SAM amino acid emission prior (recode3.20comp) has more than twice the number of free parameters compared to the default HMMER emission prior (20 and 9 component mixtures, respectively). We ran HMMER using recode3.20comp on our test. This gave an increase in performance both for global/local and local/local models, showing that the emission prior is important in explaining why SAM model building is more sensitive (Figure 2).

**Figure 3:**
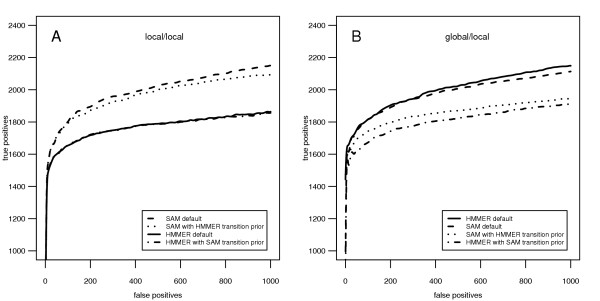
**Analysis of transition prior probabilities**. **(A) **Local/local mode. **(B) **Global/local mode. SAM and HMMER models were built using their own default transition prior, and with default transition prior from the other program. The transition priors seem to be specific to their programs as they degrade the performance of the other program.

We also investigated the role of the transition prior. This is not as straightforward since the HMMER transition prior has only seven parameters and the SAM prior has nine; the delete-insert and insert-delete transitions are non-existing in the HMMER architecture. Nevertheless, we ran HMMER with the SAM transition prior ignoring the two superfluous insert-delete parameters, and SAM with the HMMER transition prior plus the insert-delete parameters from the SAM prior. In local/local mode we could see almost no effect of using a foreign transition prior (Figure 3a), but in global/local mode the performance deteriorated considerably (Figure 3b).

The test of prior probabilities thus revealed that the SAM emission prior is an important factor to explain SAM model building superiority, while the default transition priors were program-specific for global/local mode. In all subsequent HMMER experiments, we used the SAM emission prior in order to reduce the difference in parameter settings.

### Sequence weighting

Sequence weighting involves (a) the relative weight assigned to each sequence and (b) the total weight given to all sequences as a group. While the relative weights determine the influence of one sequence relative to the others, the total weight gives the influence of the sequences vis-à-vis the prior probabilities. In addition, SAM model building involves a filter that reduces the number of training sequences such that no two sequences have more than 80% sequence identity. Excluding the filter had no important impact on SAM results (data not shown), hence the filter was removed in subsequent runs.

We analysed whether differences in sequence weighting could be a source for package-specific results. First we turned off both the relative weighting scheme and the total weight calculation in both packages. The effect of these changes is that each sequence gets a weight of 1.0, such that all sequences will be equally important and the total weight will be the number of training sequences. These changes had a negative effect on both packages, but the effect was much worse for SAM (data not shown). Two conclusions could be drawn: 1) Sequence weighting does play a major role for performance, and 2) the SAM weighting procedure is more important for performance than the HMMER weighting.

**Figure 4:**
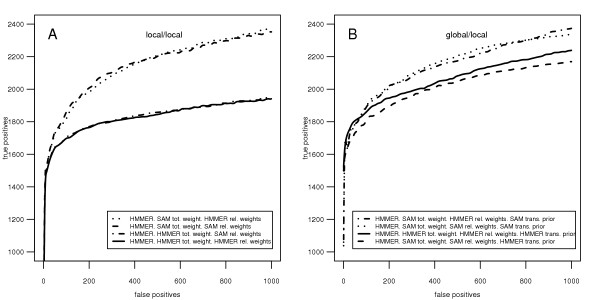
**Analysis of relative and total sequence weight calculation methods**. **(A) **Local/local mode. **(B) **Global/local mode. HMMER was run with relative and total sequence weights produced by HMMER or SAM in different combinations. In local/local mode the benefit of SAM's total weight is strong. In global/local mode, the benefit is less pronounced and dependent on using SAM's transition prior. With our implementation of SAM's "bits saved" method for total weight calculation, HMMER performed about as well as using weights estimated by SAM. SAM's recode3.20.comp emission prior was used for all model building.

Would SAM weighting work better also for HMMER? To answer this question the HMMER code was modified to read sequence weights from file, with the option to rescale those weights according to HMMER's total weight calculation. We let SAM generate weights and used them in HMMER model building. For local/local models this had a very large effect and sensitivity improved greatly when using SAM weights instead of HMMER weights (Figure 4a).

We next analysed what makes SAM weights better: the relative weighting algorithm or the total weight calculation. In order to answer this we needed to isolate the effect of the two weighting components. We let HMMER read SAM weights from file but rescaled them by the HMMER total weight; in this way HMMER was run using the SAM relative weighting algorithm but the HMMER total weight calculation. Performance dropped to a level comparable to all-HMMER weighting (Figure 4a), which indicates that the SAM total weight calculation is the crucial factor. To verify this we implemented our own version of the SAM "bits saved" method for total weight calculation in the HMMER code (see Methods). We used the SAM default target value of 0.5 bits saved relative to the background distribution. Using HMMER relative weighting and the "bits saved" method produced as good results as using SAM weights. The conclusion is that the SAM "bits saved" method for calculating the total weight is much better than the HMMER method and a main source of the difference in performance, while the schemes for relative weight calculation are essentially equivalent.

The previous tests were all done for local/local scoring. For global/local scoring the picture was less clear. Running HMMER with SAM weights in global/local mode decreased performance (Figure 4b) compared to using HMMER weights, i.e. a result opposite to what we saw for local/local mode. However, when we also added the SAM transition prior, in addition to the SAM emission prior used for all runs, the results were improved (Figure 4b). Remember that the SAM transition prior earlier proved far from optimal for global/local HMMER usage (Figure 3b). Apparently the transition prior and the total weight cannot be specified independently in order to obtain sensitive global/local HMMs.

In global/local mode, SAM sequence weights thus gave more accurate HMMER models provided they were combined with the SAM transition prior. We again split this effect into relative weighting and total weight calculation, and as for local/local scoring, the improvement was entirely due to the SAM method for total weight calculation (Figure 4b).

The total weight calculation emerges from this study as a very important component in profile HMM building. The higher the total weight, the larger will be the influence of the multiple alignment on the HMM, at the expense of the prior probabilities. Is SAM performing better because it assigns more weight or less weight to the multiple alignment, compared to HMMER? To answer this question, we investigated the output of the SAM and HMMER methods for total weight calculation on our test set of 505 Pfam families. SAM produced an average total weight of 11.8 and HMMER an average total weight of 39.8. Profile HMMs by HMMER are thus relatively more determined by the multiple alignment, while SAM gives a stronger influence to the prior probabilities. HMMER's weak performance in our test together with these numbers suggest that HMMER might overfit its models to the training data.

In conclusion, SAM sequence weighting proved more accurate than HMMER weighting. The difference was entirely due to SAM's method for total weight calculation, while the methods for relative weighting seemed to be of equivalent quality. The choice of transition prior had no influence on local/local searches. However, for global/local models, the transition prior employed and the method for calculating total weights could not be chosen independently. The best performance was obtained using SAM total weight and the SAM transition prior. However, if HMMER's transition prior was employed, the HMMER total weight calculation was more appropriate.

### Choosing match nodes

HMMER labels columns in the multiple alignment as "match" or "insert" nodes based on an automatic procedure where the overall probability of the sequences is maximised. SAM has no such algorithm and treats every column as a match column, in case nothing else is specified in the alignment. We turned off the HMMER automatic algorithm and made it assign every column to match/delete states, as is SAM default. This had a slightly negative effect on performance, suggesting that the HMMER automatic algorithm is sensible and gives some improvement (data not shown).

### Model scoring

As seen in Figure 1, HMMER model scoring is more accurate than SAM's. We believe that the principal reason for the difference lies in the used null model. Both packages calculate log-odds scores, that tell how much better the sequence matches the family-trained model than the null model. The simplest null model is based on the average amino acid frequencies in protein coding sequences. By default, HMMER and SAM use more advanced alternatives designed to compensate for the effect of biased sequence composition. This occurs when a sequence gets a relatively high score only because its overall amino acid composition is close to that of the modelled domain. HMMER compensates for biased composition by correcting the score using a second null model which is calculated as the average over all emission probabilities of the states in the target sequence's path through the model. SAM on the other hand uses the score of the reversed sequence as null model score. Unfortunately, the reversed sequence null model is compulsory for SAM's E-value calculation, hence we were unable to investigate the effect of the null model. In any case we can only improve the free HMMER code, which already seems to have the superior method.

### Comparison to earlier work

Madera and Gough carried out a similar benchmark of HMMER and SAM[20]. The authors analyzed local/local mode only and concluded that SAM was better at model building while the results for model scoring were not clear as different tests generated different results. Our study agrees with theirs on model building, but not for model scoring where our results indicate that HMMER is more accurate. From where does this discrepancy stem? Madera and Gough ran the test the way a non-expert user would, i.e. with all default settings. This means forward scoring (sum of all paths) for SAM and Viterbi scoring (single best path) for HMMER (personal communication, Martin Madera). On our test set and for both packages, forward scoring was more accurate than Viterbi scoring, but is a slower algorithm. If the authors had compared similar scoring algorithms they would most likely have concluded that HMMER scoring performs better.

## Conclusion

We have presented a comparison of the SAM and HMMER packages. SAM stands out as the better package for building HMMs; particularly so for local/local searches. SAM loses some of this advantage due to a slightly worse performing search algorithm, and for global/local mode the HMMER package was actually at par with SAM. However, if default settings are applied, SAM should be the preferred package.

We furthermore sought for the key factors in profile HMM estimation by analysing what makes SAM build more sensitive HMMs. SAM's emission prior proved clearly superior to HMMER's. The relative sequence weighting schemes of the two packages, although different, proved to perform essentially equivalently. The main effect, however, was due to how prior probabilities and multiple alignment counts are combined. The total sequence weight, which determines the degree of faith in the observed data relative to the prior probabilities, seems to be much better handled by the SAM package. It is generally correct to say that, compared to HMMER, SAM puts more belief in the prior and less in the observed alignment. Our results suggest that HMMER is overfitting models to the observed data while SAM is better utilising the Dirichlet mixture's capability to extrapolate observed amino acids to the underlying distribution.

By dissecting the importance of the different components in HMM building and scoring, we were able to combine the best features of HMMER and SAM into a modified HMMER program that is superior to both programs. The code for this and the test used in the study is freely available from the authors via ftp . Profile HMMs are used by many databases that have a large influence on genome annotation. Improvements to the profile HMM technology will therefore be of potentially large importance, which should render the results presented here valuable for many genome projects.

Looking ahead, a recent development is profile HMM – profile HMM scoring[30] which has showed significantly higher sensitivity than ordinary profile HMM to sequence searches as well as profile – profile[31,32] searches. Profile HMM – profile HMM searches can detect remote homology between two protein families. Alternatively, a multiple alignment can be constructed automatically around a single query sequence; from this a profile HMM can be built and used in a search against for example the Pfam database. The inclusion of homologs in the search improves sensitivity, and one can speculate that profile HMM – profile HMM searches gradually will out-compete profile HMM to sequence searches. Profile HMM estimation, however, is a fundamental issue also for this novel technology and we expect there is room for improvements.

## Methods

### Total weight calculation

In order to assess the importance of different methods for total weight calculation, we implemented a version of the SAM "bits saved" method in the HMMER code.

This method is unpublished by the original inventors but explained in an article by Edgar and Sjölander[33]. The number of bits saved is the relative entropy between the final HMM and an HMM defined from the Dirichlet mixture prior only (the background model), and is written as:



*P*_*D*_*(a) *is the background probability of emitting amino acid *a*, *P*_*j*_*(a) *is the emission probability vector of match state *j *in the HMM and *M *is the number of match states in the HMM. The summation is over all match states *j *and all amino acids *a*. The background probability vector *P*_*D*_*(a) *is defined by applying the Dirichlet mixture to a zero count vector. During profile HMM estimation, the total weight is adjusted iteratively until the number of bits saved matches a target entropy specified by the user.

### Programs and settings

For the comparisons in this paper we used the HMMER programs hmmbuild to build models and hmmsearch to score sequences. To build SAM models we used the w0.5 script, which is recommended for constructing models for homology detection at the superfamily level. The SAM program hmmscore was used to score sequences to SAM models. In order to achieve proper E-values, all models were calibrated using the HMMER program hmmcalibrate and the SAM hmmscore program. For scoring, we employed the Viterbi algorithm. The "forward" algorithm, which is SAM's default, generally produces better results but is much slower and hardly suitable for large scale database searches.

### Conversion between model formats

In order to isolate the performance of the build and search components of SAM and HMMER, we converted HMMs models between the two model formats. Models built by one program can thus be used for searching by both packages' scoring programs independently. Also, models from both packages can be converted to the same model format and scored using the same scoring program. For the conversion we used a program developed by Madera and Gough, with our own minor modifications. Since the original code only converts between SAM models and local/local HMMER models, we extended it to also allow conversions to global/local HMMER models.

The number of allowed transitions differs between the two model formats, which makes a complete mapping between them impossible. The conversion program handles this as follows. In SAM to HMMER conversions, the two extra SAM delete-insert transition parameters are simply omitted, causing a small information loss. In HMMER to SAM conversions, no information is lost, and converting a model from HMMER to SAM format and again back to HMMER format will re-create the original model, provided it is configured for the same score mode. According to Madera and Gough, testing indicated that the two extra SAM transitions are redundant and the information loss in SAM to HMMER conversions of no importance[34].

## Authors' contributions

MW wrote the code for the analysis, designed the test set and performed all experiments. ES participated in the design of the study. Both authors collaborated in writing the final version.

## References

[B1] Park J, Karplus K, Barrett C, Hughey R, Haussler D, Hubbard T, Chothia C (1998). Sequence comparisons using multiple sequences detect three times as many remote homologues as pairwise methods. J Mol Biol.

[B2] Hughey R, Krogh A (1996). Hidden Markov models for sequence analysis: extension and analysis of the basic method. Comput Appl Biosci.

[B3] Eddy SR (1998). Profile hidden Markov models. Bioinformatics.

[B4] Bateman A, Coin L, Durbin R, Finn RD, Hollich V, Griffiths-Jones S, Khanna A, Marshall M, Moxon S, Sonnhammer EL, Studholme DJ, Yeats C, Eddy SR (2004). The Pfam protein families database. Nucleic Acids Res.

[B5] Gough J, Karplus K, Hughey R, Chothia C (2001). Assignment of homology to genome sequences using a library of hidden Markov models that represent all proteins of known structure. J Mol Biol.

[B6] Krogh A, Mitchison G (1995). Maximum entropy weighting of aligned sequences of proteins or DNA. Proc Int Conf Intell Syst Mol Biol.

[B7] Karchin R, Hughey R (1998). Weighting hidden Markov models for maximum discrimination. Bioinformatics.

[B8] Barrett C, Hughey R, Karplus K (1997). Scoring hidden Markov models. Comput Appl Biosci.

[B9] Brown M, Hughey R, Krogh A, Mian IS, Sjolander K, Haussler D (1993). Using Dirichlet mixture priors to derive hidden Markov models for protein families. Proc Int Conf Intell Syst Mol Biol.

[B10] Sjolander K, Karplus K, Brown M, Hughey R, Krogh A, Mian IS, Haussler D (1996). Dirichlet mixtures: a method for improved detection of weak but significant protein sequence homology. Comput Appl Biosci.

[B11] Wistrand M, Sonnhammer EL (2004). Transition priors for protein hidden Markov models: an empirical study towards maximum discrimination. J Comput Biol.

[B12] Eddy SR, Mitchison G, Durbin R (1995). Maximum discrimination hidden Markov models of sequence consensus. J Comput Biol.

[B13] Wistrand M, Sonnhammer EL (2004). Improving profile HMM discrimination by adapting transition probabilities. J Mol Biol.

[B14] Mitchison GJ, Durbin R (1995). Tree-based maximal likelihood substitution matrices and hidden Markov models. Journal of Molecular Evolution.

[B15] Qian B, Goldstein RA (2003). Detecting distant homologs using phylogenetic tree-based HMMs. Proteins.

[B16] Hargbo J, Elofsson A (1999). Hidden Markov models that use predicted secondary structures for fold recognition. Proteins.

[B17] Karchin R, Cline M, Mandel-Gutfreund Y, Karplus K (2003). Hidden Markov models that use predicted local structure for fold recognition: alphabets of backbone geometry. Proteins.

[B18] Coin L, Bateman A, Durbin R (2004). Enhanced protein domain discovery using taxonomy. BMC Bioinformatics.

[B19] Coin L, Bateman A, Durbin R (2003). Enhanced protein domain discovery by using language modeling techniques from speech recognition. Proc Natl Acad Sci U S A.

[B20] Madera M, Gough J (2002). A comparison of profile hidden Markov model procedures for remote homology detection. Nucleic Acids Res.

[B21] Karplus K, Barrett C, Hughey R (1998). Hidden Markov models for detecting remote protein homologies. Bioinformatics.

[B22] Karplus K, Karchin R, Barrett C, Tu S, Cline M, Diekhans M, Grate L, Casper J, Hughey R (2001). What is the value added by human intervention in protein structure prediction?. Proteins.

[B23] Haft DH, Selengut JD, White O (2003). The TIGRFAMs database of protein families. Nucleic Acids Res.

[B24] Letunic I, Copley RR, Schmidt S, Ciccarelli FD, Doerks T, Schultz J, Ponting CP, Bork P (2004). SMART 4.0: towards genomic data integration. Nucleic Acids Res.

[B25] Gerstein M, Sonnhammer EL, Chothia C (1994). Volume changes in protein evolution. J Mol Biol.

[B26] Murzin AG, Brenner SE, Hubbard T, Chothia C (1995). SCOP: a structural classification of proteins database for the investigation of sequences and structures. J Mol Biol.

[B27] Lindahl E, Elofsson A (2000). Identification of related proteins on family, superfamily and fold level. J Mol Biol.

[B28] Brenner SE, Chothia C, Hubbard TJ (1998). Assessing sequence comparison methods with reliable structurally identified distant evolutionary relationships. Proc Natl Acad Sci U S A.

[B29] Chandonia JM, Hon G, Walker NS, Lo Conte L, Koehl P, Levitt M, Brenner SE (2004). The ASTRAL Compendium in 2004. Nucleic Acids Res.

[B30] Soding J (2005). Protein homology detection by HMM-HMM comparison. Bioinformatics.

[B31] Sadreyev R, Grishin N (2003). COMPASS: a tool for comparison of multiple protein alignments with assessment of statistical significance. J Mol Biol.

[B32] Yona G, Levitt M (2002). Within the twilight zone: a sensitive profile-profile comparison tool based on information theory. J Mol Biol.

[B33] Edgar RC, Sjolander K (2003). SATCHMO: sequence alignment and tree construction using hidden Markov models. Bioinformatics.

[B34] Madera MGJ A conversion program between SAM and HMMER. http://www.mrc-lmb.cam.ac.uk/genomes/julian/convert/descr.html.

